# The 50‐year‐old pulmonary artery catheter: the tale of a foretold death?

**DOI:** 10.1002/ehf2.12702

**Published:** 2020-04-20

**Authors:** Guillaume Leurent, Clément Delmas, Vincent Auffret, Eric Bonnefoy, Etienne Puymirat, FranÇois Roubille

**Affiliations:** ^1^ Department of Cardiology, CHU Rennes, Inserm, LTSI—UMR 1099 Univ Rennes 1 F‐35000 Rennes France; ^2^ Intensive Cardiac Care Unit, Department of Cardiology Rangueil University Hospital; INSERM 1048 Metabolic and Cardiovascular Disease Institute 31059 Toulouse France; ^3^ Department of Cardiac Critical Care Hospices Civils de Lyon Lyon France; ^4^ Department of Cardiology Assistance Publique‐Hôpitaux de Paris (AP‐HP); Hôpital Européen Georges Pompidou (HEGP) Paris France; ^5^ Université de Paris Paris France; ^6^ PhyMedExp, Université de Montpellier, INSERM, CNRS Cardiology Department, CHU de Montpellier France

It is said that the concept of the Swan–Ganz catheter came up in Dr Swan's mind in the 1960s after watching the sailboats in Santa Monica Bay being drifted by streams and winds: he assumed that a balloon‐ended catheter would be driven into the pulmonary artery by the blood flowing out of the pulmonary outflow tract, allowing a catheterization of the pulmonary artery without the aid of fluoroscopy. Following the presentation of the balloon flotation catheter, its use and its potential benefits in the *New England Journal of Medicine*,^1^ 50 years ago, pulmonary artery catheterization (PAC) promptly became a haemodynamic assessment and monitoring gold standard, particularly in the critical care setting.

Inserted from a central vein to a pulmonary artery, PAC allows measurements of right heart, pulmonary capillary wedge pressure (PCWP), and cardiac outflow. Beyond the absolute values of the recorded pressure, valuable data can be provided by the morphology of the curves. A proper analysis of those curves thus allows diagnosis of mitral regurgitation or pericardial constriction, for instance. Moreover, it offers possibility of stepped blood sampling, for measurement of central oxygenation or quantification of intra‐cardiac shunt. Last, if retained, PAC allows bedside continuous monitoring and also an individually tailored management, such as the evaluation of the haemodynamic responsiveness to a fluid challenge or an inotrope/vasopressor infusion. This opportunity of a dynamic evaluation made the PAC a theoretical perfect tool for management of critically ill patients, especially in the case of cardiogenic shock (CS). Various devices have been proposed to provide more sophisticated approaches but without further evidences for efficacy.

However, PAC has three obvious major limitations.

First is the safety concern: as an invasive tool, PAC provides potential significant complications. Besides vascular complications because of central venous catheterization (mostly catheter‐related bloodstream infection or deep vein thrombosis, pneumothorax, and vascular wound)^2^—which can be significantly decreased with the help of vascular‐echo‐guidance—pulmonary infarction/haemorrhage and ventricular arrhythmia are typical PAC‐related damages. The reported complications rate is especially high (5–10%) because patients are critically ill (haemorrhagic events in patients with anti‐thrombotic treatment, sepsis in immunocompromised patient, etc.) and the operators poorly trained.

Second, PAC use requires specific skills: because the haemodynamic situation is often complex, multifaceted, and time varying, the interpretation and analysis of the recorded values and curves can be challenging. Thus, any misinterpretation of the recorded haemodynamic data can lead to inappropriate therapy.

Last but not least is the lack of proven benefit: to date, no randomized trial has shown any improvement of the mortality outcome with a routine PAC.^3^ Importantly, this lack of proven benefit is consistently found for all the haemodynamic monitoring methods.

With this background, strong data about the use of PAC in large cohorts, during a long study period, are more than expected. In this issue of *ESC Heart Failure*, Vallabhajosyula *et al*. present 15 years' nationwide data about 364,001 admissions with CS in the setting of an acute myocardial infarction.^4^ The authors provide important data illustrating the trends in the field. The main finding is a 75% decrease of PAC use during the study period, from 13.9% in 2000 to 5.4% in 2014. Moreover, the use of a PAC identified a higher risk cohort with greater organ failure and higher in‐hospital mortality. Last, whereas in‐hospital mortality is significantly higher in the PAC cohort, it becomes comparable in the two cohorts after a propensity‐matched analysis.

Consistently, we conducted in 2020 January a survey about PAC and invasive assessment usage patterns in order to better understand the dramatic decrease of use of PAC. This survey was conducted in 32 French intensive care units (ICUs), mostly university centres (*n* = 29 centres; 87%) and level 2 ICU with on‐site interventional cardiology (*n* = 21 centres, 66%). Fifty per cent reported never using the Swan–Ganz catheter in daily practice. The main causes of non‐use of PAC monitoring were as follows: preference for echocardiographic assessment (*n* = 9, 31%) or iterative invasive haemodynamic evaluation by serial right‐heart catheterization (RHC) when considered useful (*n* = 7, 24%), absence of evidence for benefit (*n* = 5, 17%), and/or worries about PAC‐associated complications (*n* = 4, 14%). Moreover, invasive assessment is poorly used in general, because 13 centres (41%) reported never having performed intermittent or iterative RHC, whereas left ventricular end‐diastolic pressure (LVEDP) in patients with myocardial infarction is never recorded in 14 centres (44%), even in the case of low cardiac output, elevated filling pressures, or mechanical complication suspicion. Unsurprisingly, PAC monitoring is mainly adopted in patients with CS, mixed shock, and right heart failure and in patients under mechanical cardiac support (MCS), whereas iterative RHC is more frequently used in the case of heart transplantation or MCS discussion, pulmonary hypertension, or pericardial constriction suspicion. Last, the Swan–Ganz is mainly used in level 3 ICU, whereas level 2 ICU rather rely on iterative RHC.

This impressive collapse of the PAC use in the ICU can obviously be explained by its unfavourable risk–benefit ratio as compared with echocardiography (*Figure* *1*). Mostly, we can assume that non‐invasive assessment by echocardiography is the true gravedigger of the PAC: guidelines recommend an immediate echocardiography with a higher level of recommendation (I) than PAC (IIb) in the management of an acute CS.^5^ Indeed, echocardiography allows a safe and reproducible evaluation of ventricular and valvular functions and loading conditions. However, the filling pressure assessment by echocardiography (i) is operator dependent and requires specific skills as well; (ii) has not been validated or can be very challenging in many frequent situations (e.g. severe valvular heart disease, previous myocardial infarction involving basal septum and/or basal lateral wall, atrial fibrillation, left bundle branch block or pacemaker, hypertrophic cardiomyopathy, and pericardial disease); (iii) requires adequate echocardiographic window, which is sometimes tricky in overweight patients or patients under mechanical ventilation; and (iv) has a low positive predictive value for identification of patients with elevated invasive LVEDP (≥15 mmHg).^6^

**Figure 1:**
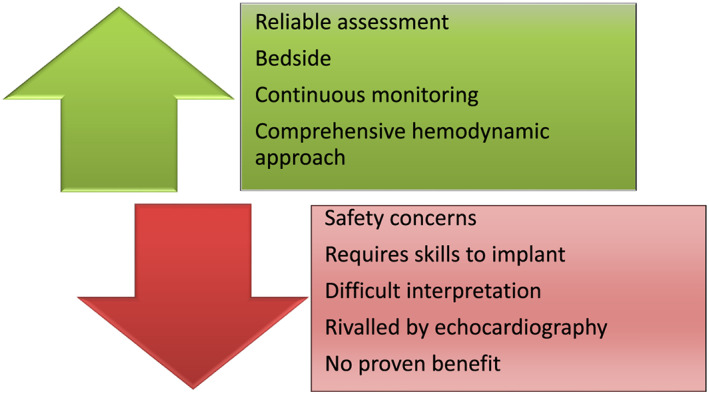
Upside and downside of pulmonary artery catheter.

Two schematic settings could probably be distinguished: on the one hand, severe patients with not too complex haemodynamic evaluation (e.g. a CS following a large anterior myocardial infarction without any other comorbidities) and, on the other hand, severe and complex situations, all the more as the haemodynamic appraisal of critically ill patients is often highly variable. Thus, a multi‐parametric modality assessment including invasive haemodynamic parameters seems important for complex haemodynamic impairments such as in a patient with a ventilator‐acquired pneumonia and extra‐corporal life support, or a severe hypotension in a patient with deep vasoplegia and severely depressed ejection fraction in the setting of a post‐cardiac arrest syndrome.

Last, this decrease of PAC use was conducted with a modification of CS definition itself, because the initial PAC‐recorded haemodynamic criteria (low cardiac index and high PCWP) have been abandoned in the last ESC definition. Because we strongly believe that the haemodynamic assessment of critically ill patients requires a multi‐parametric modality assessment, we recently proposed a new integrative definition for CS. This so‐called Frenshock definition (NCT02703038) considers all the available modalities—encompassing clinical, biological, radiological, echographic, and haemodynamic data—for assessment of the three necessary parts (low cardiac output, elevated pressures, and organ malperfusions) of the CS.^7^

In conclusion, Vallabhajosyula *et al*. report expected decrease of PAC use in ischemic CS in the USA,^4^ which seems to be a general state. However, there is a small residual place for PAC, especially in the management of the most complex patients. Therefore, student and junior doctors should still learn how to select adequately the patients benefiting from PAC, to implant PAC, and analyse collected data.

## Conflict of interest

None declared.
